# Editorial: Non-lymphoid functions of regulatory T cells in health and disease

**DOI:** 10.3389/fimmu.2023.1109245

**Published:** 2023-01-16

**Authors:** Paula D. Bos, Akihiko Yoshimura, Dipayan Rudra

**Affiliations:** ^1^ Department of Pathology, Virginia Commonwealth University School of Medicine, Richmond, VA, United States; ^2^ Massey Cancer Center, Virginia Commonwealth University School of Medicine, Richmond, VA, United States; ^3^ Department of Microbiology and Immunology, Keio University School of Medicine, Tokyo, Japan; ^4^ School of Life Science & Technology, ShanghaiTech University, Shanghai, China

**Keywords:** Treg heterogeneity, Treg tissue adaptation, lymphoid and non-lymphoid Treg function, Treg physiology, Treg origin

Over exuberant immune response can be self-destructive and is required to be efficiently controlled to avoid collateral damage. Foxp3^+^ regulatory T (Treg) cells are a subtype of CD4^+^ lymphocytes that serve this paramount purpose through mechanisms of peripheral tolerance (1). On the other hand, due to their inherent immune-regulatory properties, Treg cells are obstructive to anti-tumor immunity, and are well-recognized targets in cancer immunotherapy (2). Contrary to earlier beliefs when they were considered to be a universal immunosuppressive population, research in the last several years have led to the identification of a rather diverse and distinct pool of heterogenous Treg subsets. Phenotypically and functionally distinct Treg populations have been identified in non-lymphoid tissues and tumor sites where their physiologic significance is now well recognized under various contexts of health and disease. This series of articles in *Frontiers in Immunology* presents 12 full length review articles, 3 mini reviews, 2 original research articles and one brief research report, covering various aspects of the biology of Treg cells of non-lymphoid origin, with an overall goal of providing a comprehensive viewpoint of prominent researchers in the field for the readers of this Research Topic.

Two review articles by Lee et al. and Malko et al. provide comprehensive discussions and current updates on the overall progress on the research on non-lymphoid Treg populations in various tissues ranging from visceral adipose tissue (VAT), muscle, kidney, liver, reproductive organs, as well as barrier sites like skin, lung and intestine and the Central nervous system (CNS). The authors further provide a brief overview on the therapeutic interventions that are currently pursued in the clinic to utilize Treg depletion or promotion strategies in the context of cancer immunotherapy and tissue-specific inflammatory and autoimmune diseases. In order to exert their tissue-specific effects, Treg cells utilize a variety of tissue homing and retention strategies by which they migrate to peripheral tissue sites, respond to microenvironment specific survival signals, and exert tissue protective functions. Brull et al. covers this aspect and discuss tissue-specific adaptation strategies that shape up cellular crosstalk between Treg cells and immune and non-immune cell types, both in the context of barrier tissue environment as well as immune privileged sites like CNS.

Epigenetic reprogramming is key in establishing and maintaining Treg cell function, and likely a main player in generating their diversity. Kraj discusses how Bone Morphogenetic Protein (BMP) signaling might contribute to this epigenetic reprograming through BMP receptor BMPR1a signaling, as deletion of BMPR1a in mature Treg cells leads to increasingly more peripheral Treg cells with lower levels of Foxp3 and a naïve phenotype, including reduced suppressive function. Besides mediators of plasticity and adaptation, striking changes in Kdm6b demethylase and Cdkn1 cell cycle inhibitor were observed, suggesting a dysregulated epigenetic reprogramming.

Over the last decade, immunometabolism has evolved as a crucial area of research interconnecting the changes in metabolism to the quantity and quality of immune response. Not surprisingly, distinct metabolic adaptation strategies are also utilized by Treg cells in non-lymphoid environments and at tumor sites to ensure survival, hemostasis and tissue specific functions. The article by Yang discuss recent progress in our understanding of Treg metabolism in the context of tissue homeostasis and functions, and its implications on defining Treg-mediated and Treg-aiming novel therapeutic strategies against autoimmunity and cancer. In another review article, Lu et al. focus on downstream effector mechanism of metabolic reprogramming and metabolite mediated epigenetic modifications in Treg cells and its implications on Treg activation, differentiation and function.

Heart failure is a worldwide problem with high rates of hospitalization and mortality. Two review articles provide comprehensive summary on the function and significance of Treg cells in myocardial infarction and conditions of chronic heart failure. Myocardial infarction (MI) involves a rapid inflammatory response followed by replacement of dead myocardium with fibrous tissue. Recent studies have shown that Treg cells directly promote proliferation and survival of cardiomyocytes in MI mice by producing CST7, TNFSFL1, IL33, FGL2, MATN2, and IGF2 (3). The review article by Weiß et al. summarize recent advances on our understanding of Treg-mediated MI repair, made possible by the use of T cell receptor (TCR) transgenic mouse models with defined antigen specificity against the cardiac-specific part of the myosin heavy alpha chain MYHCA antigen (4). Contrary to MI, in chronic heart failure, Treg cells promote angiogenesis and change to a profibrotic cell type. The article by Lu et al. summarize their function in the pathogenesis of chronic heart failure and focusses on the interaction between Treg cell and their target cells, which include cells of immune (e.g., monocytes/macrophages, dendritic cells, T cells, B cells) as well as parenchymal (e.g., cardiomyocytes, fibroblasts, endothelial cells) origin.

Three articles of the Research Topic discuss Treg cells in the brain. Treg cells are abundant in the gastrointestinal tract, where microbiota induced pTreg and Treg cells of thymic origin, tTreg cells, coexist (5). The gastrointestinal tract and the central nervous system are functionally connected, providing evidence that Treg cells function along the gut-brain axis, interacting with immune cells, epithelial cells, and neurons. For example, the neurotransmitter acetylcholine activates antigen-presenting cells in the colon and may support the development of Treg cells in the gut (6). Choi et al. describe current knowledge regarding the biological role of tissue-resident Treg cells and their interactions along the gut-brain axis. Two original research articles describe their findings on brain Treg cells. Ito et al. previously showed that large numbers of Treg cells infiltrate and expand in the brain during the chronic phase after stroke and are involved in neural repair (7). IL-33 and its receptor ST2 play an important role in their proliferation upon induction of brain injury. In one study, Xie et al. demonstrated a fundamental role of IL-33/ST2 signaling on Treg cells in traumatic brain injury (TBI) model. IL-33 administration after TBI significantly reduced brain lesion size and improved neurological function, and importantly, Treg cell depletion significantly reduced the protective effect of IL-33 after TBI. Thus IL-33 and Treg cells may represent a novel immunotherapeutic strategy to improve TBI outcome. In another original research article, Yamamoto et al. made a challenging trial to create brain Treg cells *in vitro*. They co-cultured spleen-derived Treg cells with primary astrocytes or microglia to mimic the brain environment in the presence of various cytokines and growth factors. The results showed that the addition of IL-2, IL-33, and histamine to the astrocyte co-culture partially conferred characteristics similar to the brain Treg cell population. Brain Treg-like cells generated *in vitro* showed increased brain infiltration and improved pathology in stroke and Parkinson’s disease models compared with splenic Treg cells.

Skin, as a barrier organ, is heavily exposed to commensals and pathogens implicating that a fine balance between effector and immune tolerance mechanisms must exist in order to ensure proper immune homeostasis. Experimental evidence in the past several years strongly suggest that skin resident Treg cells, in addition to committing to such so called classical immune suppressive mechanisms, also take part in non-immune functions like wound healing, repair as well as hair follicle cycling (8, 9). The review article by Hajam et al. discuss the current progress in our understanding on recently discovered non-canonical unconventional functions of skin Treg cells. A different aspect of Treg-mediated unconventional functions is elaborated in recent years by using zebra fish as a model system, where Treg like (zTreg) cells have been identified, and their spectacular regenerative properties have been demonstrated in multiple organs (10). As a follow up of this study, an original brief research report by Hui et al. demonstrates the role of zTreg cells in the regeneration of zebrafish caudal fin tissue. By employing a caudal fin amputation model in zebra fish, the authors demonstrate that zTreg cells promote vigorous proliferation of blastemal cells residing underneath wound epidermis by producing insulin like growth factors Igf2a and Igf2b, consequently leading to efficient tissue regeneration.

As critical enforcers of peripheral tolerance, Treg cells contribute to cancer establishment and progression (11). Treg cell infiltration and higher ratio of Treg to other effector T cells correlate with poor prognosis in cancer patients (2). Their intrinsic ability to suppress immune responses is critical to tumor control, but our increased knowledge of the physiological function of Treg cells in tissues suggest mechanisms beyond traditional immune suppression. A collection of reviews on this topic examine the phenotype, function and therapeutic targeting of tumor Treg cells. Qiu et al. describe the interplay between factors known to control original and emerging hallmarks of cancer and the reciprocal and dynamic interactions they have with non-lymphoid Treg cells. In a short review, Gao et al. discuss how tumor cell-modulated elements of the tumor microenvironment (such as aminoacids, nucleic acids and glucose) modulate Treg cell surface molecules, cytokine production and transcriptional regulators. Moreover, they describe the potential of current immunomodulatory therapies and give examples of how their anti-tumor effects might relate to targeting different functions of the intratumoral Treg cell compartment. Sekiya presents the role of the nuclear orphan receptor family Nr4a in the differentiation and maintenance of thymic Treg cells, and the nuances in Nr4a1 and 2 function, that seem to be heightened by signals from the tumor microenvironment. Within solid tumors, colorectal cancer (CRC) presents a quite unique situation, due in part to the large environmental interface that hosts most our commensal microorganisms. CRC has been characterized by a high frequency of TH17-like Treg cells, with an increased capacity to suppress T cell proliferation *via* their reduced expression of Tcf-1, yet increased pro-inflammatory function that drives polyp growth. As a consequence, there are contradictory observations regarding Treg cell correlation with outcome. Aristin-Revilla et al. dive deep into the heterogeneity of intestinal Treg cell populations, factors that contribute to generate it, and how this might explain the paradox regarding infiltration of Treg cells and prognosis in colorectal cancer. Importantly, they describe current therapeutic efforts designed to target molecules important for CRC tumor-infiltrating Treg cells and how they are faring in clinical trials.

Pregnancy represents a complex physiological situation in which a foreign tissue is growing within another living organism. This semi-allogeneic scenario evokes tumor immunity, and Treg cells play similar roles inducing a tolerogenic environment in which these semi-foreign, invasive tissues continue to grow. Muralidhara et al. draws parallels between uterine and tumor Treg cells, and highlights the differences driven by the dynamic nature of the inflammatory environment and the mix-nature of foreign antigens. These issues are critical when considering treating cancer with anti-Treg cell therapies during pregnancy.

We edited this Research Topic to highlight and update readers on Treg cell phenotypic diversity and role in physiological functions beyond peripheral tolerance. We hope the audience benefits from recent discoveries and contributes to expand our knowledge of this growing field.

## Author contributions

All authors listed have made a substantial, direct, and intellectual contribution to the work and approved it for publication.
